# Carbapenem-Resistant *Burkholderia cepacia* Complex Isolates Carrying *bla*_NDM−1_ and *bla*_NDM−5_ in Ventilator-Associated Pneumonia Patients and Contaminated Ventilator Tubing

**DOI:** 10.1155/2024/3352135

**Published:** 2024-08-30

**Authors:** Muhammad Saeed, Farhan Rasheed, Muhammad Hidayat Rasool, Sumreen Hayat, Mohsin Khurshid

**Affiliations:** ^1^ Institute of Microbiology Government College University Faisalabad, Faisalabad, Pakistan; ^2^ Allama Iqbal Medical College and Jinnah Hospital, Lahore, Pakistan

## Abstract

Ventilator-associated pneumonia (VAP) represents an important nosocomial infection, frequently encountered in intensive care unit (ICU) settings which results in prolonged hospitals stays. The nosocomial infections caused by *Burkholderia cepacia* complex (BCC) bacteria pose a significant challenge in healthcare settings owing to their intrinsic resistance to many antibiotics. This study investigates the antimicrobial susceptibility patterns and mechanisms of carbapenem resistance among BCC bacteria from VAP patients and the ventilator tubing. The blood and respiratory specimens from patients diagnosed with VAP were collected. In addition, the ventilators were also screened for the presence of BCC bacteria. The susceptibility profiling of BCC isolates was performed against the various antimicrobial agents, and screening for acquired beta-lactamase enzymes was conducted by polymerase chain reaction. Out of the total 134 patients with BCC-associated VAP, *B. cepacia*, *Burkholderia multivorans*, and *Burkholderia cenocepacia* was 68.7% (*n* = 92), 18.7% (*n* = 25), and 12.7% (*n* = 17). Overall, the BCC isolates showed varying susceptibility to different antibiotics: 76.9% were susceptible to chloramphenicol, 76.1% to minocycline, 69.4% to meropenem, 60.4% to ceftazidime, 51.5% to trimethoprim-sulfamethoxazole, and 50% to levofloxacin. Resistance to ceftazidime (51/92, 55.4%) and meropenem (36/92, 39.1%) was exclusively observed in *B. cepacia* isolates, and all isolates of *B. multivorans* and *B. cenocepacia* were found to be susceptible to both beta-lactam drugs. Among the 134 clinical isolates, 15 were found to harbor the *bla*_NDM_ variants, that is, *bla*_NDM−1_ and *bla*_NDM−5_. All carbapenem-resistant isolates from the ventilator tubing were identified as *B. cepacia* and were found to harbor either the *bla*_NDM−1_ or the *bla*_NDM−5_ variants. The observed increase in resistance and the emergence of acquired beta-lactamases among BCC isolates highlight a concerning trend that could potentially lead to serious outbreaks.

## 1. Introduction

Ventilator-associated pneumonia (VAP) is characterized by an infection in the lung tissue of patients who have undergone mechanical ventilation for at least 48 hr [1]. Despite advancements in clinical diagnostics, the diagnosis of VAP is challenging and poses an economic burden on healthcare systems. The reported incidence of VAP varies widely, affecting 5%–40% of patients receiving invasive mechanical ventilation for more than 2 days [2]. The incidence rates vary significantly depending on factors such as the country, type of intensive care unit (ICU), and the criteria used to define VAP. While VAP is quite common in high-income countries, much higher rates are reported in lower-middle-income countries [3]. The types of microorganisms associated with VAP vary based on several factors, including the duration of mechanical ventilation, the length of hospital and ICU stays preceding the onset of VAP, the timing and cumulative use of antimicrobial agents, the local microbial environment, and the occurrence of potential epidemic events within a specific ICU [4]. Common microorganisms implicated in VAP include *Pseudomonas aeruginosa*, *Escherichia coli*, *Klebsiella pneumoniae*, and *Acinetobacter* species. Additionally, *Staphylococcus aureus* and *Burkholderia cepacia* can also play a role in VAP cases [5, 6].

*B. cepacia*, an aerobic, gram-negative, and nonfermentative bacterium commonly found in the environment, can cause serious infections in people with cystic fibrosis, certain lung diseases, or weakened immune systems [7]. Outbreaks caused by members of the *B. cepacia* complex (BCC) can be traced to a range of sources. Some recent reports have documented transmission of BCC from contaminated liquids or moist surfaces within hospital settings. Remarkably, it exhibits resilience in fluids, antiseptic solutions, and healthcare environments, persisting for extended periods [8]. BCC demonstrates intrinsic resistance to first- and second-generation cephalosporins, aminoglycosides, antipseudomonal penicillins, and polymyxins. The treatment regimens often involve medications such as levofloxacin, ceftazidime, minocycline, meropenem, or cotrimoxazole; however, resistance to these agents has also been reported [9, 10]. Carbapenem resistance in BCC is primarily attributed to the presence of an inducible class A PenB beta-lactamase. Although a study has reported instances of high-level ceftazidime resistance due to the presence of several beta-lactamase-encoding genes, as well as meropenem resistance encoded by *bla*_KPC_ genes, in BCC strains isolated from soil [11], it is worth mentioning that these acquired carbapenemases have not been reported from clinical isolates of BCC thus far.

In 2022, a series of BCC infections were reported among multiple patients suffering from VAP at a tertiary care hospital in Lahore, Pakistan. Notably, these BCC isolates exhibited high-level resistance to meropenem. In response to this, we initiated a comprehensive surveillance program to monitor BCC infections. This program included the analysis of isolates obtained from respiratory specimens and blood samples from VAP patients across various hospital wards. Additionally, we conducted screenings of ventilator tubing, both the inspiratory and expiratory limbs, to detect the presence of carbapenem-resistant (CR) BCC. This report investigates the antimicrobial resistance patterns of VAP caused by BCC in a tertiary care hospital within Lahore, Pakistan. Furthermore, it explores the concerning emergence of *bla*_NDM_ variants among these BCC isolates.

## 2. Methods

### 2.1. Study Settings

This prospective observational study was conducted from June 2022 to May 2023 at Jinnah Hospital, which is a tertiary care hospital in Lahore, the capital of Punjab, Pakistan. Informed consent was duly acquired from the patients or their immediate family members, and the study received approval from the Ethical Review Committee of the Government College University Faisalabad, Pakistan, and Ethical Review Board of the Jinnah Hospital, Lahore, Pakistan.

Inclusion criteria: Patients of all ages who have been admitted to the hospital and placed on a ventilator for at least 48 hr before developing symptoms have provided written informed consent to participate in the study and had a positive culture test result.

Exclusion criteria: Patients who have had previous episodes of VAP during the current hospitalization or have received antibiotic treatment within 48 hr before the onset of symptoms. Patients with significant comorbid conditions that could confound the study results, such as severe immunosuppression (e.g., due to HIV/AIDS and chemotherapy) or terminal illness. Patients with incomplete medical records and those transferred from another hospital or healthcare facility where they might have already acquired VAP.

VAP was diagnosed based on the age-specific criteria established by the Centers for Disease Control and Prevention (CDC) [2, 12]. This diagnosis involved the presence of radiographic evidence, indications of new or progressing lung infiltrates, and at least three of the following criteria:

1. Fever (> 38°C) or hypothermia (<36°C)
2. Abnormal white blood cell count (≥15,000/mm^3^ or ≤4,000/mm^3^)
3. Purulent tracheal secretions or a change in the character of tracheal secretions
4. Cough or shortness of breath
5. Apnea or rapid breathing
6. The presence of rales or bronchial breath sounds
7. Abnormal heart rate (<100 beats/min or > 170 beats/min) in infants
8. Deteriorating gas exchange

Confirmation of VAP diagnosis was established through positive culture results from the respiratory specimens including tracheal secretions (TS), endotracheal tube tip (ETT), bronchial washings (BW), bronchoalveolar lavage (BAL), and endotracheal wash (EW) with a bacterial count > 10^4^ CFU/mL [12].

### 2.2. Isolation and Identification

The blood (237) and respiratory specimens (171) were collected by the attending critical care physician using aseptic procedures. Blood cultures were processed using the automated culture system BD BACTEC™ 9050 (Becton and Dickinson Microbiology System, MD, USA). The BACTEC vials were inoculated with the blood of patients following the manufacturer's guidelines and were incubated until a positive signal was detected. Upon observing a positive signal, the bottles were removed from the instrument, and gram staining of the smears prepared from the positive blood culture bottles was performed [13] followed by streaking on blood agar (Oxoid, UK), MacConkey agar (Oxoid, UK), and chocolate agar (Oxoid, UK) [14]. Samples from the respiratory tracts of patients were collected and diluted in sterile normal saline, and the resulting dilution was inoculated onto the aforementioned agar plate in accordance with previously established protocols [15, 16]. These plates were incubated at a temperature of 37°C for a period ranging from 24 to 48 hr. Moreover, the samples were collected from the inspiratory and expiratory limbs of the ventilators using sterile swabs and sterile saline solution. These samples were then cultured on blood agar (Oxoid, UK), MacConkey agar (Oxoid, UK), and chocolate agar (Oxoid, UK) supplemented with 16 *µ*g/mL meropenem. The identification of bacterial isolates was carried out through the utilization of the VITEK®2 system (bioMérieux SA, Marcy-l'Etoile, France). Further confirmation was carried out by MALDI-TOF VITEK MS (bioMérieux) as per manufacturer guidelines. All the isolated strains were preserved in Brain Heart Infusion (BHI) broth (Oxoid, UK) with glycerol at a temperature of −80°C. Prior to conducting tests, they were cultured on BHI agar (Oxoid, UK).

### 2.3. Antimicrobial Susceptibility Profiling

To assess the antimicrobial susceptibility of the isolates, disc diffusion assays (DDA) for various antimicrobial agents, including ceftazidime, meropenem, minocycline, and trimethoprim-sulfamethoxazole were conducted followed by broth microdilution (BMD) assays. For levofloxacin and chloramphenicol, only BMD assays were carried out. The antibiotic discs and powders were purchased from local vendors and manufactured by Oxoid, UK, and Sigma–Aldrich, USA, respectively. The outcomes of both DDA and BMD assays were analyzed in accordance with the Clinical and Laboratory Standards Institute (CLSI, 2022) guidelines. Quality control was maintained through the utilization of *E. coli* strains (ATCC-35218 and ATCC-25922) and *P. aeruginosa* (ATCC-27853) [17, 18].

### 2.4. Genetic Screening for Beta-Lactam Resistance

Bacterial DNA was extracted from the colonies using the FavorPrep™ Genomic DNA Extraction Kit (Favorgen Biotech Corporation, Pingtung, Taiwan) following the instructions provided by the manufacturer in the kit. The extracted DNA was then analyzed on a 1% agarose gel, stained with ethidium bromide, and visualized using UV transilluminator. The extracted DNA was stored at −20°C until further use.

To identify genes encoding extended-spectrum beta-lactamases (ESBLs) such as *bla*_TEM_, *bla*_SHV_, *bla*_CTXM_, and carbapenemases including *bla*_OXA−23_, *bla*_OXA−24_, *bla*_OXA−48_, *bla*_OXA−51_, *bla*_OXA−58_, *bla*_IMP_, *bla*_VIM_, *bla*_SIM_, *bla*_GIM_, *bla*_SPM_, *bla*_NDM_, and *bla*_KPC_, PCR was employed with primers specified in *Supplementary table 1*. Briefly, the reaction mixture (50 *µ*L) comprised 25 *µ*L of 2X PCR Master Mix (Thermo Fisher Scientific, Massachusetts, USA), 1 *µ*L of each primer (10 *µ*M) (Macrogen, South Korea), and 1.5 *µ*L of the DNA sample. The PCR process was carried out using a T100 Thermal Cycler (Bio-Rad Laboratories, California, USA) with the following parameters: initial denaturation at 95°C for 3 min, followed by 35 cycles of 95°C for 30 s (cyclic denaturation), variable temperature (*Supplementary table 1*) for 30 s (annealing), 72°C for 30 s (extension), and a final extension step at 72°C for 12 min. Subsequently, DNA sequencing was employed to validate the PCR products. The full-length *bla*_NDM_ gene was amplified, and subsequent sequencing was carried out to confirm the presence of variants [17, 18].

## 3. Results

### 3.1. Patients Characteristics

This study involved 134 cases of VAP caused by BCC between June 2022 and May 2023. Due to the typically low incidence of BCC-associated VAP [6], the sudden rise in BCC infections was classified as an outbreak. The median age of the patients infected with BCC was 45 ± 29.2 years (ranging from 1 to 88 years), with a majority being male (*n* = 85; 63.4%). Their average hospital stay duration was 12.5 ± 6.6 days (ranging from 3 to 14 days), while the time of BCC diagnosis occurred at an average of 7.4 ± 3.5 days (ranging from 4 to 28 days) after patients were placed on ventilators. The specimens from which BCC bacteria were obtained included blood from 72 (53.7%) patients, BW and TS from 17 (12.7%) patients each, BAL from 13 (9.7%) patients, ETTs from nine (6.7%) patients, and EW from six (4.5%) patients as shown in Table 1.

### 3.2. Resistance Patterns of Isolates

The study identified 134 BCC isolates from patients with VAP. Of these isolates, 68.7% (*n* = 92) were identified as *B. cepacia*, and 18.7% (*n* = 25) and 12.7% (*n* = 17) were identified as *B. multivorans* and *B. cenocepacia*, respectively. Additionally, 20 isolates from the ventilator tubing were identified as *B. cepacia*. The overall antibiogram analysis of BCC isolates revealed the following results: 103 (76.9%) were susceptible to chloramphenicol, 102 (76.1%) to minocycline, 93 (69.4%) to meropenem, 81 (60.4%) to ceftazidime, 69 (51.5%) to trimethoprim-sulfamethoxazole, and 67 (50%) to levofloxacin (Table 2).

**Figure 1:**
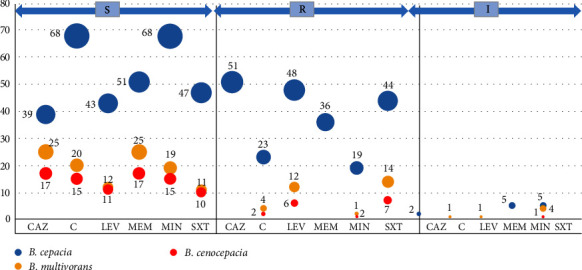
Bubble plot showing phenotypic antibiotic resistance pattern of BCC clinical isolates. The *Y*-axis shows the relative abundance of isolates, while the *X*-axis shows the antimicrobial agents, that is, CAZ, ceftazidime; C, chloramphenicol; LEV, levofloxacin; MEM, meropenem; MIN, minocycline; SXT, trimethoprim-sulfamethoxazole.

**Figure 2:**
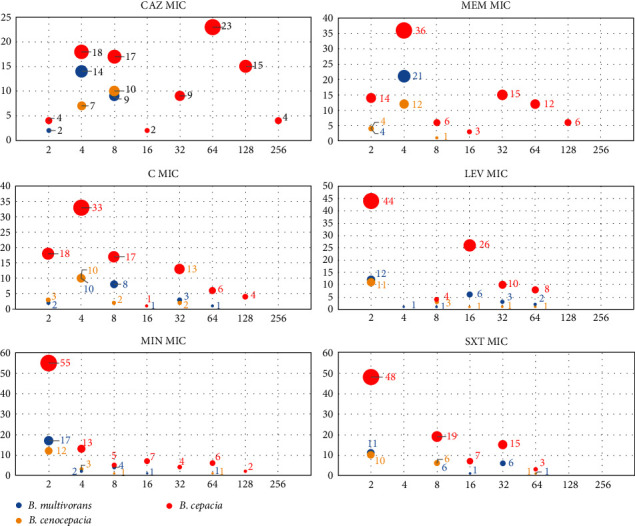
Bubble graph showing the distribution of minimum inhibitory concentrations (MICs) of various antimicrobial agents against BCC isolates from VAP patients. The *Y*-axis shows the number of isolates, while the *X*-axis shows the MIC values. CAZ, ceftazidime; MEM, meropenem; C, chloramphenicol; LEV, levofloxacin; MIN, minocycline; SXT, trimethoprim-sulfamethoxazole.

Among the 92 *B. cepacia* isolates from VAP patients, 51 (55.4%) showed resistance to ceftazidime, whereas all isolates of *B. multivorans* and *B. cenocepacia* were found to be susceptible. In the case of meropenem, 36 (39.1%) *B. cepacia* isolates exhibited resistance, while all of the *B. multivorans* and *B. cenocepacia* isolates were susceptible to meropenem. As for chloramphenicol, the resistance rates for *B. cepacia*, *B. multivorans*, and *B. cenocepacia* were 25%, 16%, and 11.8%, respectively. Regarding levofloxacin, 48 (52.2%) *B. cepacia* isolates were resistant, along with 12 (48%) of *B. multivorans* and 6 (35.3%) of *B. cenocepacia*. The resistance rates to minocycline among *B. cepacia*, *B. multivorans*, and *B. cenocepacia* were 20.7%, 8%, and 5.9%, respectively. For trimethoprim-sulfamethoxazole, the resistance rates were quite similar: 47.8% for *B. cepacia*, 56% for *B. multivorans*, and 41.2% for *B. cenocepacia* (Figure 1 and Table 2). For the BCC isolates from VAP patients, the MIC_50_ and MIC_90_ values for ceftazidime were 8 and 128, respectively, encompassing a range from 2 to ≥256 *µ*g/mL. Similarly, for meropenem, the MIC_50_ and MIC_90_ values were 4 and 64, respectively, with a range spanning from 1 to 128 *µ*g/mL (Figure 2). The comprehensive distribution of minimum inhibitory concentrations (MICs) for various antimicrobial agents against the BCC isolates is presented in Table 3.

The 20 *B. cepacia* isolates from ventilator tubing exhibited a similar resistance profile to chloramphenicol, levofloxacin, minocycline, and trimethoprim-sulfamethoxazole. In contrast, all of these isolates showed resistance to ceftazidime and meropenem (Table 4).

### 3.3. Genotypic Characterization of Carbapenem Resistance

**Figure 3:**
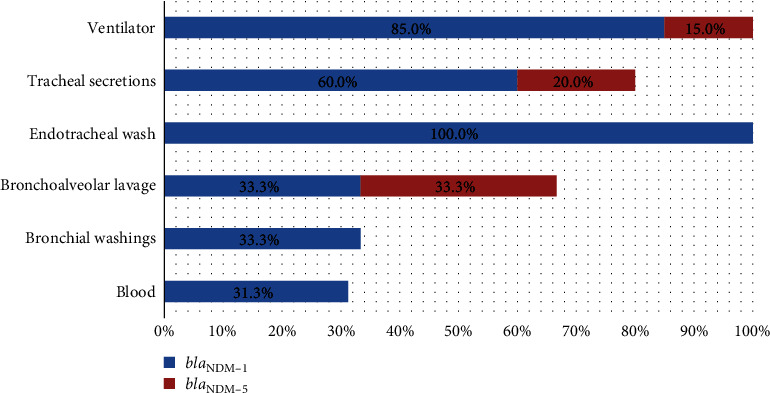
Percentage distribution of *bla*_NDM_ variants among carbapenem-resistant *B. cepacia* isolates from VAP patients and ventilator tubing.

Carbapenem resistance was exclusively observed among the *B. cepacia* isolates. Among these 92 *B. cepacia* isolates, 36 showed carbapenem resistance, and 15 (41.6%) were found to harbor the *bla*_NDM_ gene (*Supplementary table 2*). Among these, two isolates were carrying the *bla*_NDM−5_, and the remaining 13 were carrying the *bla*_NDM−1_ variant. Notably, none of the *B. multivorans* and *B. cenocepacia* isolates carried the *bla*_NDM_ gene. Furthermore, none of the BCC isolates harbored any of the tested ESBLs, oxacillinases, or metallo-beta-lactamases (MBL) genes screened in this study, except for *bla*_NDM_. All the 20 CR *B. cepacia* isolates recovered from ventilators that harbored the *bla*_NDM_ gene, with the majority (17 isolates) carrying *bla*_NDM−1_ and three isolates carrying *bla*_NDM−5_ (Figure 3).

## 4. Discussion

In healthcare settings, multidrug-resistant (MDR) gram-negative bacteria (GNB) cause a variety of infections that have emerged as a significant challenge [19]. Despite being present in the environment, the BCC organisms are comparatively an infrequent cause of infections in humans. The infections are particularly common among immunocompromised individuals, such as patients with cystic fibrosis or patients in ICUs; therefore, these infections can lead to substantial morbidity and mortality [20]. BCC bacteria can survive and grow in hospitals which makes them a frequent culprit in hospital outbreaks. Contaminated items like antiseptics, disinfectants, and nebulizer solutions have all been linked to BCC outbreaks [21]. This study is one of the first to identify the species of BCC involved in VAP and to evaluate their antimicrobial resistance patterns, including the presence of the acquired carbapenem resistance gene *bla*_NDM_, in Pakistan.

The carbapenem resistance and detection of the *bla*_NDM_ gene are important as these acquired carbapenemases that had not been previously reported in clinical BCC isolates anywhere in the world. There is a notable scarcity of information regarding the involvement of *B. cepacia* in healthcare-associated infections in Pakistan. In a recent report from Pakistan, BCC bacteria isolates were predominantly obtained from blood cultures, followed by respiratory specimens, which included bronchoalveolar lavage, endobronchial washings, and sputum, whereas only a small number of isolates were recovered from body fluids. However, the BCC isolates were not identified to the species level in the study. The isolates displayed antibiotic resistance rates of 3.1% for minocycline, 6.2% for cotrimoxazole, 11.6% for levofloxacin, 17.6% for meropenem, and 28.16% for ceftazidime. In comparison, we found that 76.9% BCC isolates were susceptible to chloramphenicol, 76.1% to minocycline, 69.4% to meropenem, 60.4% to ceftazidime, 51.5% to trimethoprim-sulfamethoxazole, and 50% to levofloxacin. This shows higher resistance rates to the recommended antibiotics compared to the previous study from Pakistan [22]. Antimicrobial susceptibility patterns exhibit variations that are attributable to several factors. Overall antibiotic prescribing practices and usage within healthcare settings significantly impact these patterns. Further, inherent genetic differences among bacterial strains can lead to varying baseline levels of resistance. Moreover, horizontal gene transfer facilitates the rapid acquisition of resistance genes by bacteria, allowing them to gain resistance from neighboring bacteria or their environment. Notably, hospitals often exhibit higher rates of antibiotic use and antibiotic-resistant bacteria compared to community settings. This phenomenon can be attributed to factors such as the presence of immunocompromised patients who are more susceptible to infections and require frequent antibiotic treatment, as well as the routine use of invasive procedures that can introduce bacteria into the bloodstream [23, 24].

Studies have demonstrated that *B. cepacia* inherently exhibits resistance to several classes of antibiotics, including aminoglycosides, ampicillin, amoxicillin, ticarcillin, piperacillin, amoxicillin-clavulanate, amoxicillin-sulbactam, polymyxin B, colistin, ertapenem, and fosfomycin [25]. Among the BCC bacteria, resistance to beta-lactam antibiotics, such as ceftazidime, is primarily driven by the upregulation of class A beta-lactamases, that is, PenA and PenB [26]. In addition to the presence of these inducible chromosomal beta-lactamases, several studies have reported that resistance to beta-lactam antibiotics can also arise from mechanisms that reduce the access of the drug to its target, thereby giving rise to the hypothesis that multiple synergistic mechanisms contribute to this resistance [26, 27]. So far, acquired ESBLs and carbapenemases have not been documented in clinical settings among BCC bacteria. Nevertheless, a study has documented the presence of acquired beta-lactamase-encoding genes in the BCC isolated from Brazilian soils. Notably, among the ESBLs, *bla*_SHV_ was prevalent, and *bla*_KPC_ was also identified in three isolates of soil origin [11].

The strains harboring *bla*_NDM_ pose a significant threat as this gene confers resistance to carbapenems, which are considered drugs of last resort for the treatment of nosocomial infections. The Asian continent serves as the primary reservoir of *bla*_NDM_ producers, accounting for approximately 58.15% of the total, primarily concentrated in China and India. In Europe, there are roughly 16.8% of the total *bla*_NDM_ producers, with the variant being most prevalent in countries such as, Azerbaijan, Romania, Poland, Bulgaria, France, Italy, Germany, Turkey, Greece, Ukraine, Serbia, Croatia, London, and Ireland [28].

This study marks the initial documentation of *bla*_NDM_ in *B. cepacia* isolates. It is imperative to underscore the significance of these acquired carbapenemases, particularly *bla*_NDM_, which plays a pivotal role in conferring resistance to carbapenem antibiotics. This is crucial due to its widespread dissemination across diverse Enterobacteriaceae species on a global scale [29]. The limitation of this study is the absence of epidemiological analysis as we did not conduct multilocus sequence typing (MLST) due to financial constraints. Moreover, it would be valuable to explore plasmids carrying these genes in *B. cepacia* strains, along with other bacteria from the same hospital which could shed light on the transmission dynamics of the *bla*_NDM_ gene as early studies from Pakistan have already reported the presence of the *bla*_NDM_ gene in Enterobacterales and *Acinetobacter* isolates [17, 30, 31]. Culture-based detection methods may not identify all microbes present in tested environments. Molecular methods using metagenomic DNA directly can help avoid the biases associated with culturing. To gain a more comprehensive understanding of the prevalence and dissemination mechanisms of these acquired beta-lactamases among clinical isolates particularly in the BCC bacteria in Pakistani hospitals, it is important to implement further surveillance programs.

## 5. Conclusions

In summary, the increased resistance to the already diminished antibiotic options for the BCC isolates from VAP patients, the emerging antibiotic resistance genes especially the metallo-beta-lactamases, that is, *bla*_NDM_ in BCC clinical isolates, may counteract antimicrobial-resistant bacteria in the future. Previous studies have already revealed that contaminated solutions employed in patient care activities can lead to substantial outbreaks. This study underscores the potential ramifications of BCC bacterial contamination in ventilators and its impact on critically ill patients, especially those in ICUs. In developing countries like Pakistan, where ventilators are often used for multiple patients without rigorous disinfection, it is essential to establish and adhere to proper disinfection procedures. Alternatively, the use of new tubing for each patient is highly recommended to ensure safety and prevent the spread of infections. It is imperative to promptly conduct a thorough epidemiological investigation of such clusters to pinpoint the source and implement effective outbreak control measures.

## Figures and Tables

**Table 1:** Clinical characteristics of patients in this study.

Variable	Number (%)
Age	
Median (years)	45 ± 29.3
Gender	
Male	85 (63.4)
Female	49 (36.6)
Marital status	
Married	83 (61.9)
Unmarried	51 (38.1)
Day of reporting	
Mean (days)	7.4 ± 3.5
Length of stay	
Mean (days)	12.5 ± 6.6
Specimens	
Bronchoalveolar lavage	13 (9.7)
Blood	72 (53.7)
Bronchial washings	17 (12.7)
Endotracheal tube tip	9 (6.7)
Endotracheal wash	6 (4.5)
Tracheal secretions	17 (12.7)
Antibiotic usage	
Carbapenems	122 (91)
Colistin	93 (69.4)
Vancomycin	103 (76.9)
Fluoroquinolones	35 (26.1)

**Table 2:** Antimicrobial susceptibility patterns of BCC isolates from VAP patients.

Antimicrobial agents	BCC isolates (*n* = 134)						*B. cepacia* (*n* = 92)						*B. multivorans* (*n* = 25)						*B. cenocepacia* (*n* = 17)					
	S		I		R		S		I		R		S		I		R		S		I		R	
	*n*	%	*n*	%	*n*	%	*n*	%	*n*	%	*n*	%	*n*	%	*n*	%	*n*	%	*n*	%	*n*	%	*n*	%
Ceftazidime	81	60.4	2	1.5	51	38.1	39	42.4	2	2.2	51	55.4	25	100	—	—	—	—	17	100	—	—	—	—
Meropenem	93	69.4	5	3.7	36	26.9	51	55.4	5	5.4	36	39.1	25	100	—	—	—	—	17	100	—	—	—	—
Chloramphenicol	103	76.9	2	1.5	29	21.6	68	73.9	1	1.1	23	25	20	80	1	4	4	16	15	88.2	—	—	2	11.8
Levofloxacin	67	50	1	0.7	66	49.3	44	47.8	—	—	48	52.2	12	48	1	4	12	48	11	64.7	—	—	6	35.3
Minocycline	102	76.1	10	7.5	22	16.4	68	73.9	5	5.4	19	20.7	19	76	4	16	2	8	15	88.2	1	5.9	1	5.9
Trimethoprim-sulfamethoxazole	69	51.5	—	—	65	48.5	48	52.2	—	—	44	47.8	11	44	—	—	14	56	10	58.8	—	—	7	41.2

**Table 3:** Distribution of MICs of various antimicrobial agents against BCC isolates from VAP patients.

Antimicrobial agents	MIC_50_	MIC_90_	MIC (*µ*g/mL)									
			≤0.5	1	2	4	8	16	32	64	128	≥256
Ceftazidime	8	128	—	—	6	39	36	2	9	23	15	5
Meropenem	4	64	—	4	18	69	7	3	15	12	6	—
Chloramphenicol	4	32	—	—	23	53	27	2	18	7	4	—
Levofloxacin	2	32	—	30	37	1	8	33	14	11	—	—
Minocycline	2	32	—	5	79	18	10	8	4	8	2	—
Trimethoprim-sulfamethoxazole	2/38	32/608	—	—	69	—	31	8	21	5	—	—

Trimethoprim-sulfamethoxazole was used in a 1 : 19 ratio with a concentration range of 0.5/9.5 to 256/4864 *µ*g/mL, respectively.

**Table 4:** Antimicrobial susceptibility profiling and distribution of MICs of various antimicrobial agents against BCC isolates from ventilator tubing.

Antimicrobial agents	Susceptible *n* (%)	Resistant *n* (%)	MIC (*µ*g/mL)									
			≤0.5	1	2	4	8	16	32	64	128	≥256
Ceftazidime	—	20 (100)	—	—	—	—	—	—	—	10	7	3
Meropenem	—	20 (100)	—	—	—	—	—	—	—	13	7	—
Chloramphenicol	14 (70)	6 (30)	—	—	7	6	1	—	2	2	2	—
Levofloxacin	11 (55)	9 (45)	—	2	9	—	2	4	—	3	—	—
Minocycline	18 (90)	2 (10)	—	—	15	3	—	—	—	1	1	—
Trimethoprim-sulfamethoxazole	9 (45)	11 (55)	—	—	9	—	4	—	5	2	—	—

## Data Availability

All the information supporting our conclusions and the relevant references are included in the manuscript. The datasets used and analyzed in the current study are also accessible through the corresponding author.

## Supplementary Materials

Supplementary 1 Table 1: primers and conditions for amplification of beta-lactamases genes.

Supplementary 2 Table 2: distribution of acquired beta-lactamases and their corresponding MICs among carbapenem-resistant *B. cepacia* isolates.
