# Supplementary material for: Carbapenem-Resistant Burkholderia cepacia Complex Isolates Carrying blaNDM−1 and blaNDM−5 in Ventilator-Associated Pneumonia Patients and Contaminated Ventilator Tubing
Source: Transbound Emerg Dis. 2024 Aug 30;2024:3352135. doi: 10.1155/2024/3352135 (PMC12016991; doi:10.1155/2024/3352135)
Supplement: Supplementary 1 — Table 1: primers and conditions for amplification of beta-lactamases genes. [file 3352135.f1.docx]

**Table S1: Primers and conditions for amplification of beta-lactamases genes**

| Genes | Primers | Sequence (5’ to 3’) | Aimed Product (bp) | Annealing Temperature |
| --- | --- | --- | --- | --- |
| *bla*_TEM_ | TEM-F | TCAACATTTCCGTGTCG | 860 | 56 |
|  | TEM-R | CTGACAGTTACCAATGCTTA |  |  |
| *bla*_SHV_ | SHV-F | ATGCGTTATATTCGCCTGTG | 896 | 56 |
|  | SHV-R | AGATAAATCACCACAATGCGC |  |  |
| *bla*_CTXM_ | CTXMU-F | ATGTGCAGYACCAGTAARGT | 593 | 52 |
|  | CTXMU-R | TGGGTRAARTARGTSACCAGA |  |  |
| *bla*_OXA-48_ | OXA-48-F | TTGGTGGCATCGATTATCGG | 744 | 52 |
|  | OXA-48-F | GAGCACTTCTTTTGTGATGGC |  |  |
| *bla*_IMP_ | IMP-F | GGAATAGAGTGGCTTAAYTC | 232 | 50 |
|  | IMP-R | TCGGTTTAAYAAAACAACCACC |  |  |
| *bla*_VIM_ | VIM-F | GATGGTGTTTGGTCGCATA | 390 | 50 |
|  | VIM-R | CGAATGCGCAGCACCAG |  |  |
| *bla*_SPM_ | SPM-F | AAAATCTGGGTACGCAAACG | 271 | 52 |
|  | SPM-R | ACATTATCCGCTGGAACAGG |  |  |
| *bla*_GIM_ | GIM-F | TCGACACACCTTGGTCTGAA | 477 | 50 |
|  | GIM-R | AACTTCCAACTTTGCCATGC |  |  |
| *bla*_SIM_ | SIM-F | TACAAGGGATTCGGCATCG | 570 | 50 |
|  | SIM-R | TAATGGCCTGTTCCCATGTG |  |  |
| *bla*_NDM_ | NDM-F | CACCTCATGTTTGAATTCGCC | 984 | 52 |
|  | NDM-R | CTCTGTCACATCGAAATCGC |  |  |
| *bla*_KPC_ | KPC-F | CGTCTAGTTCTGCTGTCTTG | 798 | 50 |
|  | KPC-R | CTTGTCATCCTTGTTAGGCG |  |  |
