# Supplementary material for: Carbapenem-Resistant Burkholderia cepacia Complex Isolates Carrying blaNDM−1 and blaNDM−5 in Ventilator-Associated Pneumonia Patients and Contaminated Ventilator Tubing
Source: Transbound Emerg Dis. 2024 Aug 30;2024:3352135. doi: 10.1155/2024/3352135 (PMC12016991; doi:10.1155/2024/3352135)
Supplement: Supplementary 2 — Table 2: distribution of acquired beta-lactamases and their corresponding MICs among carbapenem-resistant B. cepacia isolates. [file 3352135.f2.docx]

**Table S2: Distribution of Acquired Beta-Lactamases and Their Corresponding MICs Among Carbapenem-Resistant *B. cepacia* Isolates**

| **Source** | **BCC isolate** | **MIC** | | | | | | ***bla*_NDM_ variant** |
| --- | --- | --- | --- | --- | --- | --- | --- | --- |
|  |  | **CAZ** | **MEM** | **C** | **LEV** | **MIN** | **SXT** |  |
| Bronchial washings | *B. cepacia* | 64 | 32 | 32 | 16 | 8 | 8 | - |
| Bronchial washings | *B. cepacia* | 64 | 32 | 64 | 32 | 16 | 32 | - |
| Bronchial washings | *B. cepacia* | 128 | 64 | 32 | 16 | 64 | 8 | *bla*_NDM-1_ |
| Bronchial washings | *B. cepacia* | 64 | 32 | 32 | 16 | 2 | 32 | - |
| Bronchoalveolar lavage | *B. cepacia* | 64 | 128 | 64 | 64 | 128 | 32 | *bla*_NDM-1_ |
| Endotracheal Wash | *B. cepacia* | 128 | 128 | 2 | 1 | 2 | 32 | *bla*_NDM-1_ |
| Bronchial washings | *B. cepacia* | 32 | 64 | 4 | 1 | 1 | 2 | - |
| Blood | *B. cepacia* | 128 | 128 | 2 | 2 | 4 | 2 | *bla*_NDM-1_ |
| Blood | *B. cepacia* | 32 | 32 | 4 | 64 | 8 | 32 | - |
| Blood | *B. cepacia* | 256 | 128 | 2 | 2 | 2 | 32 | *bla*_NDM-1_ |
| Blood | *B. cepacia* | 64 | 64 | 32 | 2 | 2 | 2 | *bla*_NDM-1_ |
| Tracheal secretions | *B. cepacia* | 64 | 64 | 64 | 16 | 4 | 32 | *bla*_NDM-5_ |
| Endotracheal tube tip | *B. cepacia* | 128 | 32 | 8 | 16 | 2 | 32 | - |
| Blood | *B. cepacia* | 128 | 16 | 2 | 2 | 16 | 2 | - |
| Tracheal secretions | *B. cepacia* | 64 | 64 | 128 | 2 | 2 | 8 | *bla*_NDM-1_ |
| Tracheal secretions | *B. cepacia* | 64 | 128 | 128 | 32 | 4 | 8 | *bla*_NDM-1_ |
| Blood | *B. cepacia* | 32 | 32 | 8 | 16 | 2 | 2 | - |
| Bronchial washings | *B. cepacia* | 64 | 64 | 4 | 16 | 2 | 8 | *bla*_NDM-1_ |
| Blood | *B. cepacia* | 64 | 32 | 2 | 1 | 2 | 2 | - |
| Blood | *B. cepacia* | 32 | 16 | 2 | 16 | 2 | 2 | - |
| Bronchial washings | *B. cepacia* | 128 | 64 | 4 | 2 | 2 | 2 | *bla*_NDM-1_ |
| Tracheal secretions | *B. cepacia* | 256 | 64 | 4 | 16 | 4 | 64 | *bla*_NDM-1_ |
| Bronchoalveolar lavage | *B. cepacia* | 32 | 128 | 4 | 2 | 2 | 16 | - |
| Blood | *B. cepacia* | 64 | 64 | 4 | 64 | 2 | 2 | *bla*_NDM-1_ |
| Blood | *B. cepacia* | 32 | 64 | 8 | 2 | 2 | 2 | - |
| Bronchial washings | *B. cepacia* | 128 | 32 | 8 | 2 | 2 | 2 | - |
| Bronchoalveolar lavage | *B. cepacia* | 64 | 64 | 8 | 8 | 2 | 2 | *bla*_NDM-5_ |
| Blood | *B. cepacia* | 64 | 32 | 4 | 8 | 2 | 2 | - |
| Blood | *B. cepacia* | 128 | 32 | 32 | 8 | 32 | 8 | - |
| Blood | *B. cepacia* | 64 | 16 | 8 | 2 | 2 | 2 | - |
| Blood | *B. cepacia* | 128 | 64 | 4 | 16 | 2 | 64 | *bla*_NDM-1_ |
| Blood | *B. cepacia* | 256 | 32 | 4 | 1 | 2 | 2 | - |
| Tracheal secretions | *B. cepacia* | 128 | 32 | 4 | 2 | 2 | 2 | - |
| Blood | *B. cepacia* | 32 | 32 | 4 | 2 | 2 | 2 | - |
| Endotracheal tube tip | *B. cepacia* | 64 | 32 | 64 | 64 | 2 | 32 | - |
| Bronchial washings | *B. cepacia* | 64 | 32 | 32 | 16 | 4 | 8 | - |
| Ventilator | *B. cepacia* | 64 | 128 | 64 | 64 | 128 | 32 | *bla*_NDM-1_ |
| Ventilator | *B. cepacia* | 128 | 128 | 2 | 1 | 2 | 32 | *bla*_NDM-1_ |
| Ventilator | *B. cepacia* | 128 | 128 | 2 | 2 | 4 | 2 | *bla*_NDM-1_ |
| Ventilator | *B. cepacia* | 256 | 128 | 2 | 2 | 2 | 32 | *bla*_NDM-1_ |
| Ventilator | *B. cepacia* | 64 | 64 | 32 | 2 | 2 | 2 | *bla*_NDM-5_ |
| Ventilator | *B. cepacia* | 64 | 64 | 128 | 2 | 2 | 8 | *bla*_NDM-1_ |
| Ventilator | *B. cepacia* | 64 | 64 | 4 | 64 | 2 | 2 | *bla*_NDM-1_ |
| Ventilator | *B. cepacia* | 64 | 64 | 4 | 8 | 2 | 2 | *bla*_NDM-5_ |
| Ventilator | *B. cepacia* | 128 | 64 | 32 | 16 | 64 | 8 | *bla*_NDM-1_ |
| Ventilator | *B. cepacia* | 128 | 64 | 2 | 1 | 2 | 32 | *bla*_NDM-1_ |
| Ventilator | *B. cepacia* | 128 | 128 | 2 | 2 | 4 | 2 | *bla*_NDM-1_ |
| Ventilator | *B. cepacia* | 256 | 128 | 2 | 2 | 2 | 32 | *bla*_NDM-1_ |
| Ventilator | *B. cepacia* | 64 | 64 | 64 | 2 | 2 | 2 | *bla*_NDM-1_ |
| Ventilator | *B. cepacia* | 64 | 64 | 128 | 2 | 2 | 8 | *bla*_NDM-1_ |
| Ventilator | *B. cepacia* | 64 | 64 | 4 | 16 | 2 | 8 | *bla*_NDM-1_ |
| Ventilator | *B. cepacia* | 128 | 128 | 4 | 2 | 2 | 2 | *bla*_NDM-1_ |
| Ventilator | *B. cepacia* | 256 | 64 | 2 | 16 | 4 | 64 | *bla*_NDM-5_ |
| Ventilator | *B. cepacia* | 64 | 64 | 4 | 64 | 2 | 2 | *bla*_NDM-1_ |
| Ventilator | *B. cepacia* | 64 | 64 | 8 | 8 | 2 | 2 | *bla*_NDM-1_ |
| Ventilator | *B. cepacia* | 128 | 64 | 4 | 16 | 2 | 64 | *bla*_NDM-1_ |
